# Commentary: PIRs Mediate Innate Myeloid Cell Memory to Nonself MHC Molecules

**DOI:** 10.3389/fimmu.2021.721344

**Published:** 2021-10-14

**Authors:** Zhenlong Cai, Rui Xing, Jing Liu, Feiyue Xing

**Affiliations:** ^1^ Department of Immunobiology, Institute of Tissue Transplantation and Immunology, Ministry of Education (MOE) Key Laboratory of Tumor Molecular Biology, Jinan University, Guangzhou, China; ^2^ Ministry of Education (MOE) Key Laboratory of Protein Sciences, Tsinghua-Peking Center for Life Sciences, School of Life Sciences, Tsinghua University, Beijing, China; ^3^ Department of Prosthodontics, Jinan University School of Stomatology, Guangzhou, China

**Keywords:** MHC-1, PIR-A, Ly49, differentiation, specific innate immune memory

Major histocompatibility complex class I (MHC-I) molecules on the surface of all nucleated cells can present antigenic peptides derived from endogenous proteins to priming CD8^+^ T cells. Normally, antigens from the extracellular environment are not involved in the MHC-I-mediated pathway. Antigen-presenting cells (APCs), such as dendritic cells and macrophages, can collect exogenous proteins through endocytosis and then present antigens by not only conventional MHC-II but also MHC-I molecules, a process known as cross-presentation (1). For CD8^+^ T cells, MHC-I molecules are not only a priming signal that mediates cellular immune response to antigens, but are also required for the formation of the generation of immune memory (2). However, it was recently shown that pre-stimulated innate immune cells react more intensely to previously exposed pathogens than non-stimulated cells through epigenetic modification, suggesting that innate immune cells also display memory-like behaviors called trained immunity (3). Surprisingly, the latest study provided strong evidence that MHC-I molecules participate in specific innate immune memory formation of myeloid cells and natural (NK) cells (4, 5), which is entirely different from pattern recognition receptor (PRR)-mediated non-specific innate immune memory (**Figure 1a**).

Dai et al. in *Science* found for the first time that the paired immunoglobulin-like receptor-A (PIR-A) expressed on mast cells, B cells, myeloid lineage cells, and dendritic cells in mice could promote graft rejection *via* direct binding to MHC-I molecules on allografts when mice immunized with allogeneic splenocytes were rechallenged with the allogeneic ones from the same source donor (H-2 type). In sharp contrast, these mice did not mount heightened rejection when rechallenged with third-party grafts (different H-2 type). This memory-like rejection of grafts could last for several weeks and was only attributed to the clonal expansion of Ly6C^hi^ monocytes and macrophages after the initial stimulation. Blocking PIR-A from recognizing MHC-I with a specific antibody can inhibit the specific memory of myeloid cells and improve the transplant outcome in mice (4). Similarly, mouse NK cells can also acquire a memory response through binding of their Ly49 receptors Ly49C, Ly49I, and Ly49D to MHC-I molecules. Ly49 receptors belong to a C-type lectin-like superfamily, which mediates NK cell functions in an inhibitory manner except Ly49H and Ly49D (6). Ly49C and Ly49I (Ly49C/I) can directly recognize peptides presented by MHC-I to enhance antigen-specific responses of liver-homing CXCR6^+^ NK cells to the same or similar antigen after these cells are pre-sensitized. This is known as an adaptive-like immune memory. By contrast, Ly49D can interact with its preferred ligand H-2d in an antigen-specific manner (**Figure 1b**). Ly49D-mediated memory may lead to further clonal expansion of DAP10^+^ NK cells, similar to the PIR-A-mediated memory (5).

**Figure 1:**
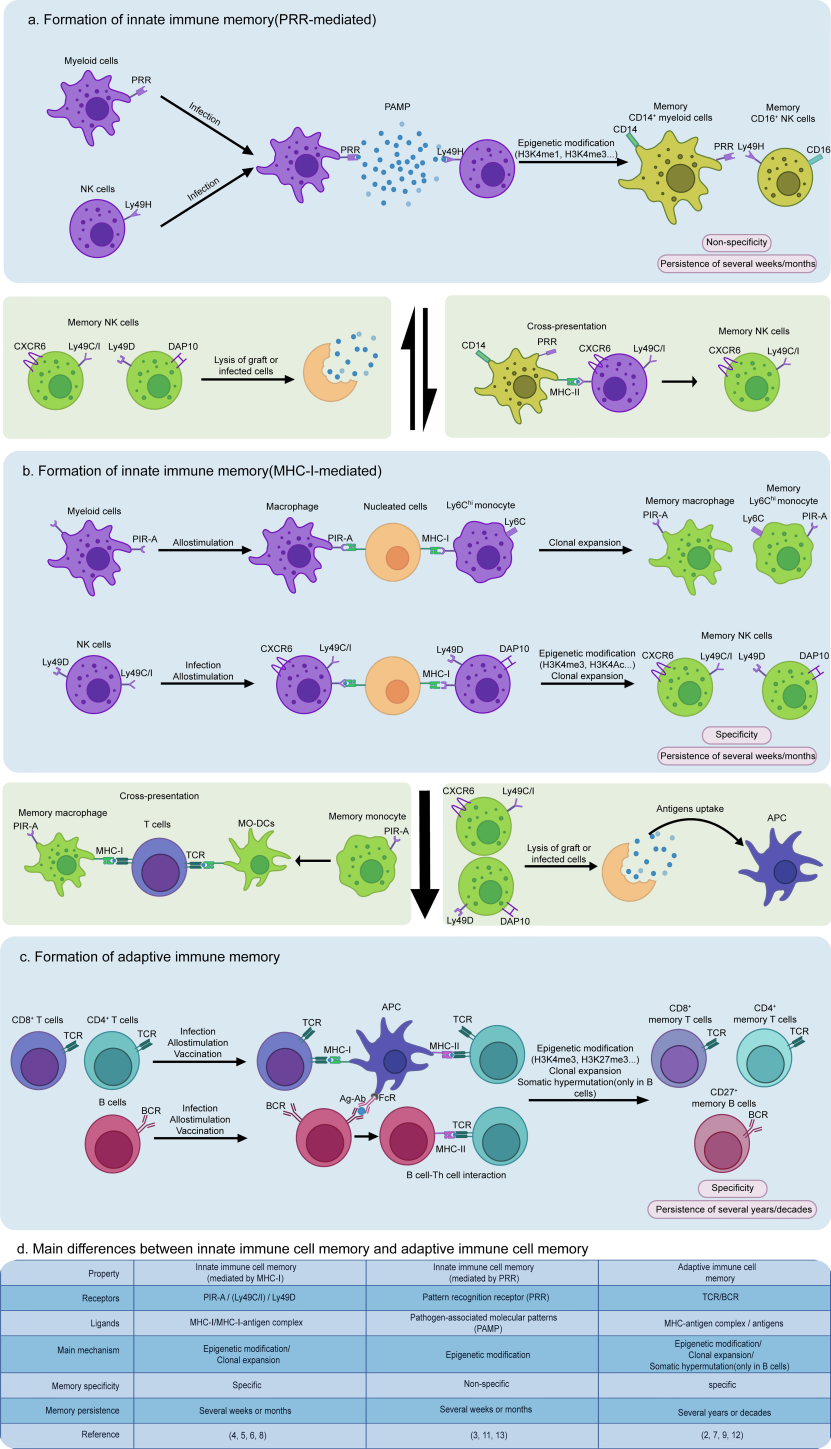
Formation and interaction of non-specific or specific innate immune memory and adaptive immune memory cells. **(a)** Innate immune cells can form non-specific memories by pattern recognition receptor (PRR) binding to pathogen-associated molecular patterns (PAMPs) from the extracellular environment, resulting in a rapid response to the same or similar antigens *via* epigenetic modification, such as H3K4me1, H3K4me3, and H3K9me2, and metabolic reprogramming, such as enhanced glycolysis and cholesterol synthesis. Moreover, during the secondary infection, memory macrophages induced by PRRs can collect and present antigens *via* cross-presentation to trigger antigen-specific memory formation of NK cells expressing Ly49C/I. **(b)** In contrast to PRR-mediated non-specific innate immune memory, MHC-I-mediated specific innate immune memory is a “cellular interaction-dependent” mechanism due to its dependence on MHC-I expressed by all nucleated cells, playing a more important role in graft rejection and killing infected cells. **(c)** Similarly, adaptive immune memory is also characterized by a “cell-dependent” manner, requiring antigen-presenting cells (APCs) to provide MHC-I/II or FcγR initial signaling to prime T cells and B cells. **(d)** The main differences between MHC-I or PRR-mediated innate immune cell memory and adaptive immune cell memory are concisely featured.

Both mouse NK and myeloid cell-mediated memory responses are related to MHC-I molecules but have marked distinctions in addition to their common specificity. There are five different isoforms among PIR-A family members in mice, including PIR-A1, PIR-A2, PIR-A3, PIR-A4, and PIR-A5, and most myeloid cells only express one of these isoforms (4). Different from expressions of PIR-A, NK cells can express more than one type of Ly49 receptor on their membrane, meaning that multiple Ly49-mediated memory mechanisms exist in one single NK cell to induce immune memory (6). Moreover, each PIR-A isoform is able to only bind one type of MHC-I, such as PIR-A3 which specifically combines with H-2Dd. Yet, Ly49C/I can interact with multiple MHC-I molecules H-2Db, H-2Kb, or H-2Dd (4, 6). Myeloid cells exhibit an increased PIR-A level in an “expanding” state that occurs in the cell cycle, antigen presentation, or allograft rejection, indicating that PIR-A expression is dynamically regulated (4). In contrast to the antigen-dependent Ly49C/I-mediated memory of NK cells, the selective myeloid cells will undergo a remarkable expansion after their expressed PIR-A “captures” its H-2 ligand, independent of whether MHC-I molecules have bound antigenic peptides (**Figure 1b**). Additionally, the expression of Ly49 by NK cells is stable, maybe due to “missing-self” function of MHC-I-deficient cells that cannot inhibit NK cells (4, 5). Neither PIR-A nor Ly49 is expressed in human NK and myeloid cells. Both of these cell types express killer cell immunoglobulin-like receptor (KIR) and leukocyte immunoglobulin-like receptor (LILR), respectively, to function as mouse PIR-A and Ly49 homologues.

MHC-I-mediated immune memory is quite distinct in cell phenotypes, memory formation mechanisms, and functional properties from PRR-mediated innate immune memory and TCR/BCR-mediated adaptive immune memory. PRRs interact directly with pathogen-associated molecular patterns (PAMPs) β-glucan, LPS and flagellin from the extracellular environment, while PIR-A or Ly49 binds MHC-I on the membrane of nucleated cells. MHC-I-mediated memory is specific, more resembling the adaptive immune memory (**Figure 1c**). By contrast, T and B cells require APCs to trigger their activation and adaptive immune memory formation directly *via* MHC- or FcR-antigen presentation on their membrane, while PRR- or MHC-I-mediated innate immune memory does not rely on antigen presentation by other cells except adaptive hepatic memory from Ly49C/I NK cells (**Figure 1**). Furthermore, the receptors involved in the innate immune memory do not undergo gene rearrangement that is shared by TCR and BCR (7).

Once a bacteria or virus invades host cells or once a graft is delivered into a host body, MHC-I from infected cells or grafts is recognized by PIR-A or Ly49, but not PRR on the surface of macrophages or NK cells, indicating that MHC-I-mediated memory plays a more important role in graft rejection. Furthermore, MHC-I-mediated memory improves interactions among innate immune cells because most innate immune cells can also express MHC-I on their membranes. For instance, NK cells can acquire an adaptive-like immune memory *via* Ly49C/I binding to a MHC-I-antigenic peptide complex from macrophages (8). Hereby, we propose that macrophages might gain memory *via* PRR binding to PAMP and then transmit this memory to NK cells expressing Ly49C/I by direct cross-presentation. In the meantime, the lysate of grafts or infected cells by MHC-I-induced memory innate immune cells can also stimulate innate immune cells to form PRR-mediated memory (**Figures 1a, b**).

This discovery of a specific innate immune cell memory indeed blurs the classical boundary between both innate and adaptive immune memories and reshapes our views on better recognition of a much closer immune regulation network. In our opinion, the immune network is bilateral and innate immune memory not only facilitates development of adaptive immune memory but also is conversely influenced by adaptive immune memory. For example, memory myeloid cells display more vigorous and rapid responses than non-memory myeloid cells when facing the same or similar antigens, becoming more effective APCs to boost CD4^+^ or CD8^+^ T cell deviation. In some cases, memory monocytes in mice can directly differentiate into monocyte-derived dendritic cells (Mo-DCs) (4). On the other hand, memory NK cells kill grafts or infected cells faster than non-memory NK cells, promoting antigen uptake by follicular dendritic cells to prime T and B cells (9). In the meantime, memory innate immune cells can act as a more powerful “bridgehead” to prevent spread of infection before the activation of adaptive immune cells. When the memory of myeloid cells fades, the antigen exposure from lysis of host cells by effector CD8^+^ T cells will stimulate activation of myeloid cells and in turn help them to regain their memory. In addition, the aid of effector CD8^+^ T cells is also required for priming the memory formation of alveolar macrophages (10).

Collectively, the finding by Dai et al. has a strong impact on the definition of traditional innate immune memory, greatly enriching the connotation of innate immune memory but also advancing our understanding of differentiation of non-specific or specific innate immune memory cells and their correlation with adaptive immune cell memory (**Figure 1d**). This discovery of MHC-I-mediated specific innate immune cell memory could lead to novel therapeutic approaches. Specific memory innate immune cells or PIR-A/Ly49 might be targeted for attenuating allograft rejection or autoimmune diseases. For example, self- or transplantation antigen-specific memory NK cells mediated by Ly49C/I could be inactivated *in vitro* and then used as a cellular vaccine *in vivo* to relieve some autoimmune diseases or allograft rejection. Tumor antigen-specific memory NK cells mediated by Ly49C/I could be induced *in vitro* and exclusively expanded *in vivo* to convert into effector NK cells or to awaken accompanying cells to attack the corresponding tumor. Attractive issues with great challenge remain to be settled in the future. Since the formation of innate immune memory mediated by PRR is usually influenced by epigenetic modification, we raise the possibility that there are potential epigenetic, metabolic, and functional differences between myeloid cells/NK cells acquiring memory *via* PIR-A/Ly49 or PRR/Ly49 signaling pathway. Obviously, solving these questions and exploring new receptors with potential to initiate non-specific and specific innate immune cell memories will provide a broader and deeper insight into the complicated immune memory network and assist man to reunite these distinct types of memories to orchestrate an entire immune response.

## Author Contributions

FX conceived of this commentary. ZC and FX drafted it. RX and JL revised the manuscript. All authors contributed to the article and approved the submitted version.

## Funding

This work was supported by the National Natural Science Foundation of China (grant numbers 81172824), and Guangzhou City Science and Technology Program Synergistic Innovation Major Project (grant number: 201604020146) to FX.

## Conflict of Interest

The authors declare that the research was conducted in the absence of any commercial or financial relationships that could be construed as a potential conflict of interest.

## Publisher’s Note

All claims expressed in this article are solely those of the authors and do not necessarily represent those of their affiliated organizations, or those of the publisher, the editors and the reviewers. Any product that may be evaluated in this article, or claim that may be made by its manufacturer, is not guaranteed or endorsed by the publisher.
